# Seasonal benthic species composition linked to coastal defense structures (CDS) in Kuala Nerus, Terengganu, Malaysia

**DOI:** 10.7717/peerj.16203

**Published:** 2023-11-14

**Authors:** Nur Fazne Ibrahim, Muzzalifah Abd Hamid, Mohd Fadzil Mohd Akhir, Meng Chuan Ong, Wan Izatul Asma Wan Talaat, Izwandy Idris

**Affiliations:** 1 Institute of Oceanography and Environment, Universiti Malaysia Terengganu, Kuala Nerus, Terengganu, Malaysia; 2 Faculty of Science and Marine Environment, Universiti Malaysia Terengganu, Kuala Nerus, Terengganu, Malaysia; 3 South China Sea Repository and Reference Centre, Institute of Oceanography and Environment, Universiti Malaysia Terengganu, Kuala Nerus, Terengganu, Malaysia

**Keywords:** Coastal defense structure, Kuala Nerus, Benthic ecosystem, Benthos, Breakwaters, Monsoon

## Abstract

**Background:**

The natural hydrodynamic process of Kuala Nerus, Terengganu, has changed since the extension of Sultan Mahmud Airport runway in 2008. Consequently, severe coastal erosion has occurred in the area, particularly during the northeast monsoon season (NEM). Numerous types of coastal defense structures (CDS) have been constructed to protect the coastline. Despite the loss of esthetic values, the effect of CDS construction on marine organisms in the area remains unknown. Hence, this study aims to assess the ecological aspects of macrobenthic compositions at the CDS area of Kuala Nerus, Terengganu, based on the differences between the southwest (SWM) and northeast (NEM) monsoon seasons.

**Methods:**

Macrobenthos were collected from the sediment in July (SWM) and December 2021 (NEM) using the Ponar grab at 12 substations from five sampling stations.

**Results:**

The density of macrobenthos was higher in SWM (48,190.82 ind./m^2^) than in NEM (24,504.83 ind./m^2^), with phylum Mollusca recording the highest species composition (60–99.3%). The macrobenthos species had a low to moderate level of diversity (H’ = 1.4–3.1) with the species were almost evenly distributed (J’ = 0.2–0.8). Windward substations exhibited coarser grain sizes (38.56%–86.84%), whereas landward substations exhibited very fine grain sizes (44.26%–86.70%). The SWM season recorded a higher organic matter content (1.6%–6.33%) than the NEM season (0.4%–3.1%). However, metal concentrations in the surface sediment were within the safe range and permissible limits for both seasons, inferring that the macrobenthos composition was unaffected.

**Discussion:**

This study demonstrated that the CDS associated with the monsoon system has controlled the hydrodynamics and nearshore sedimentary processes in the Kuala Nerus coastal zone, thereby affecting the macrobenthos population, in terms of richness and density. The ecological and energetic effects of the coastal structures in different seasons have resulted in a more significant result, with the SWM exhibiting a higher macrobenthos composition than the NEM.

## Introduction

In the tropical region of the east coast of Peninsular Malaysia, the coastlines are directly exposed to the strong winds and dynamic coastal processes of the South China Sea (SCS) (Mohd Salim et al., 2018). The negative effects of annual northeast monsoon (NEM) season exposure have been well counterbalanced by various types of coastal defense structures (CDSs), which serve as the first line of defense against strong winds, waves, and storm surges.

Coastal defense structure (CDS) is straightforwardly defined as structures that armor coastlines from natural coastal hazards such as tsunamis, typhoons, flooding, and erosion (Bulleri & Chapman, 2010). These structures consist of conventional and nonconventional structures (Anton et al., 2019). The conventional structures are further subdivided into soft (beach fill, dunes, marshes, and bioengineered) and hard (seawalls, riprap revetment, breakwaters, jetties, groins, and bulkheads) structures, with the hard structures being preferred by engineers as they are nonerodable and durable (Anton et al., 2019). The nonconventional armored structures are also known as the “living shorelines,” comprising natural and organic elements, such as wetland plantings, coral reefs, and shellfish reefs along the shoreline (Smyth et al., 2022).

Along the coastlines of Peninsular Malaysia, dykes (often called “coastal bunds” in Southeast Asia), revetments, and breakwaters were commonly constructed to prevent tidal flooding and protect reclaim land (Rashidi et al., 2021). These structures are significantly expanded and common on the west coast of Peninsular Malaysia. However, on the east coast with less common structures, there is a district named Kuala Nerus, Terengganu, with a population of 233,000 in an approximately 397.52 km^2^ area (Kuala Nerus Special Area Plan, 2022). This district, which is bordered by the South China Sea (SCS) and partially surrounded by Redang Island, has been the most populous district along the coast, with the exposure to strong wave action and erosion, particularly during the annual NEM season (Mohd Salim et al., 2018). The CDS of groynes, parallel breakwaters, and rip rap revetments were installed in response to the erosion that occurred after the completion of the runway extension at Sultan Mahmud Airport. The CDS has been proven to have successfully controlled the erosion rate. However, there is still ongoing shoreline erosion moving northward, possibly due to a recirculating current and a coastal structure that acts as a buffer.

Installing coastal structures has contributed to protecting coastal areas from erosion and reducing the risk of flooding in low-lying areas, thereby saving human residences and assets (Rashidi et al., 2021). The CDS also offers a spectacular 180-degree panoramic view that attracts people for sightseeing and recreational purposes, such as leisure fishing activities and stargazing at night. The daily and nightly exchange of land and sea breezes in the coastal structure areas has provided a peaceful mind for people and significantly reduces stress. CDS are increasingly recognized as artificial reefs that support abundant and diverse marine communities in coastal areas and play an important role in coastal ecology. The coastal structures can affect the macrobenthos assemblages primarily in different ways: (1) altering the hydrodynamic regime and physical sedimentary characteristics; (2) modifying the distribution and composition of the available food sources; (3) altering the interactions between different components of the food web (Holzhauer et al., 2020).

Consequently, among the hidden negative effects of the CDS are (1) the deterioration of water quality in the nearby area, particularly during the preconstruction process (Hashish et al., 2012); (2) the destruction of the original sedimentary habitat of the marine biota due to heavy sedimentation (Scyphers, Powers & Heck, 2015; Afghan et al., 2020; Martin et al., 2005); (3) irreversible and permanent loss of mainly benthic communities due to the placement of hard structures on the soft-bottom seafloor (Anton et al., 2019).

As a crucial component and reliable indicator in marine ecosystems, the marine macrobenthos plays a vital role in creating a productive and stable marine environment, maintaining the natural energy flow and nutrient balance in the ecosystems, linking the marine food web organisms and acting as a bioindicator species to detect pollution in an environment (Chowdhury et al., 2020; Carcedo et al., 2021; Wang et al., 2021). The soft-bottom organisms are susceptible to changes in species composition, abundance, and trophic structure due to transforming natural habitats into artificial habitats (Holzhauer et al., 2020).

In addition to the environmental variables and physical mechanisms of the CDS, the heavy metal pollution’s toxicity, persistence, and bioaccumulation characteristics can adversely impact benthic organisms (Boudaya et al., 2019). Pollution, as one of the most common anthropogenic pressures in marine environments, particularly in the coastal area, can contribute to the degradation of the marine ecosystem by altering species diversity, composition, assemblage pattern, and ecosystem function of the community (Dreujou et al., 2021; Hu et al., 2019; Roe et al., 2020). For instance, an increase in mercury (Hg) contamination in the Ria de Aveiro coastal lagoon (Portugal) has caused a decrease in the total abundance and species richness of macrobenthos (Nunes et al., 2008). In Incheon North Harbour (Korea) and the coastal zone south of Sfaz (Tunisia), a decrease in species diversity was detected as the distance from the pollution source decreased (Ryu et al., 2011; Mosbahi et al., 2019). In addition to the changes in species composition and assemblage pattern, heavy metal pollution can affect the traits of macrobenthos. In a highly polluted area, organisms typically have a shorter life span, poor mobility, feed on deposits (where the heavy metals bond to), and are more tolerant to organic matter. In contrast, in less polluted areas, they possess a longer life span, higher mobility, and higher sensitivity to organic matter (Dong et al., 2021).

In Malaysia, studies on the effects of coastal armoring on the physical and biological environment have received less scientific and conservation-focused management attention. However, despite the lack of scientific studies, the extent of coastal alteration is continuing and expanding. Thus, this study hypothesized that the coastal structures would alter the physical environment, affecting the abundance, survival, and diversity of benthic organisms in the area. This study had two main goals: (1) examine the effects of CDS on the benthic community in Kuala Nerus coastal area during monsoonal seasons; (2) relate the benthic community with seasonal environmental data.

## Materials and Methods

### Sampling design

**Figure 1:**
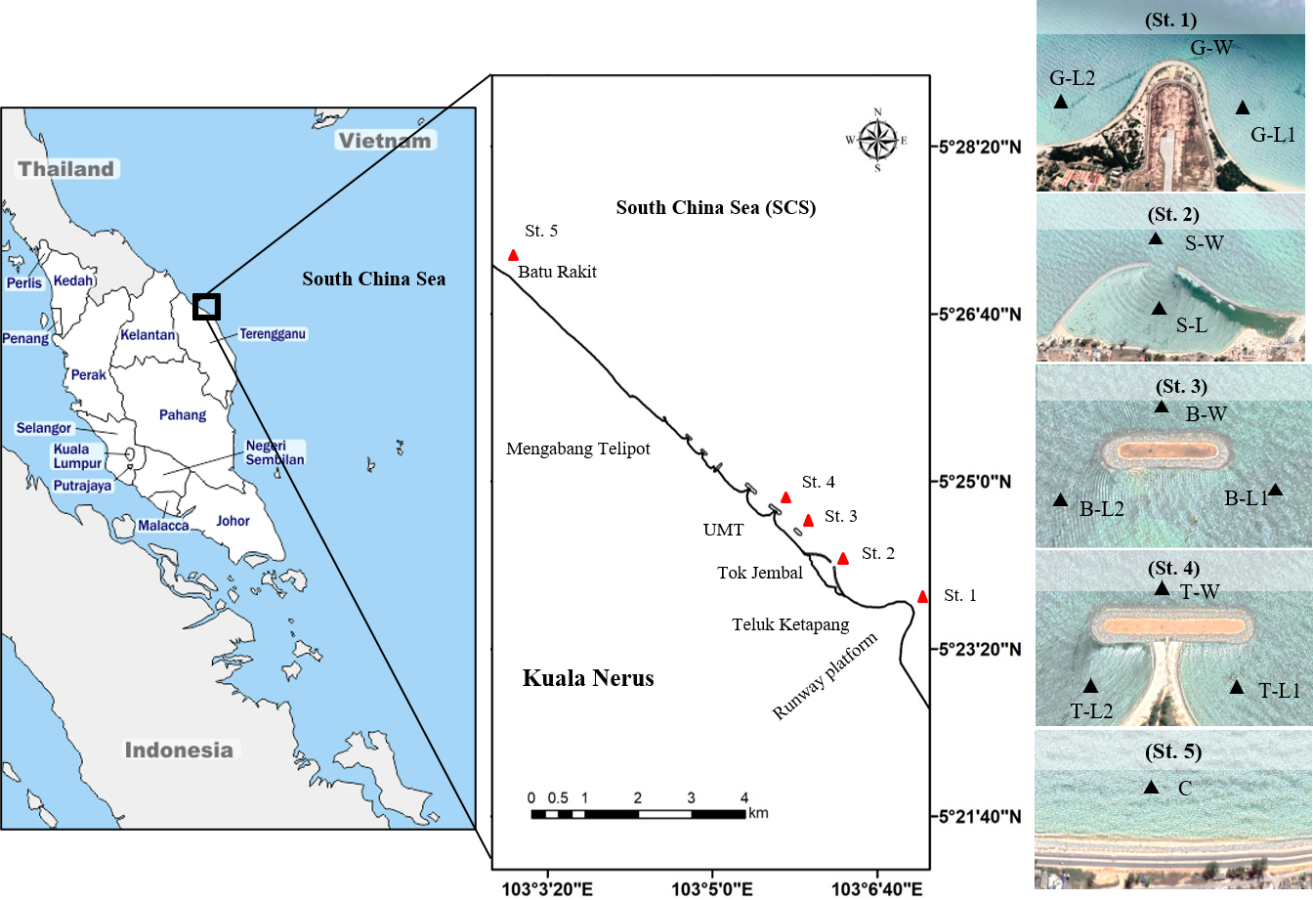
Map of study sites along Kuala Nerus coastal area. Map of Peninsular Malaysia shows the location of Kuala Nerus, Terengganu (left) and five main stations nearby the coastal defence structure along Kuala Nerus coastal area (middle and right). St. 1. Groyne (G), St. 2. Semi enclosed jetty type breakwater (S), St. 3. Breakwater without tombolo (B), St. 4. Breakwater with tombolo (T), St. 5. Control station (C) = Batu Rakit. L = Landward; W = Windward; 1 = Right side of CDS; 2 = Left side of CDS.

This study was performed in Kuala Nerus coastal area, specifically from the runway platform of Sultan Mahmud Airport (5°23′25.7”N, 103°06′59.9”E) going northward to Batu Rakit (5°27′09.0”N, 103°02′58.4”E), with the presence of three types of CDS (groyne, semi-enclosed jetty-type breakwater, and parallel breakwater—with and without tombolo) and a control station (Fig. 1, Table 1). Samples were collected during two main Asian monsoon seasons: July 2021- southwest monsoon season (SWM) and December 2021- northeast monsoon season (NEM), subjected to suitable tides, weather, and sea conditions. Along the coast of Kuala Nerus, 12 substations of the five main sampling stations with CDS were chosen and coded as follows: “station”—“substation”—“1” or “2.” The station codes are as follows: Groyne (G), semi-enclosed jetty-type breakwater (S), breakwater without tombolo (B), breakwater with tombolo (T), and control station (C). Codes for substations are windward (W) and landward (L). Windward substations refer to exposed areas directly facing the sea, whereas landward substations refer to sheltered areas facing the land. Numbers 1 and 2 are assigned to the right and left sides of the groyne and parallel breakwater, with and without tombolo, respectively. At each CDS station, samples were collected at three substations, representing the windward and landward sides of the CDS, except in semi-enclosed jetty-type breakwaters- with two substations. These sampling substations were marked using a Global Positioning System (GPS) (Table 1). The control station (without CDS) was located at Batu Rakit, approximately 3.3 nm from the last station with CDS. All sampling stations and substations were chosen based on the physical characteristics of the study sites, including the flow of the water current and longshore drift, which were expected to influence sediment types and adaptation of diverse marine organisms.

**Table 1:** Geographical location and category of 12 substations from five main stations of different coastal defence structures.

**Stations**	**Codes**	**Latitude (N)**	**Longitude (N)**	**Category**
(1) Groyne (G) = Runway platform airport	G-L1	5°23′25.7″	103°06′59.9″	Landward
	G-W	5°23′47.5″	103°07′04.3″	Windward
	G-L2	5°23′58.0″	103°06′26.6″	Landward
(2) Semi-enclosed jetty type breakwater (S)	S-L	5°24′08.2″	103°06′06.6″	Windward
	S-W	5°24′12.6″	103°06′15.5″	Landward
(3) Parallel breakwater without tombolo (B)	B-L1	5°24′21.6″	103°05′56.7″	Landward
	B-W	5°24′32.4″	103°05′54.2″	Windward
	B-L2	5°24′32.0″	103°05′45.7″	Landward
(4) Parallel breakwater with tombolo (T)	T-L1	5°24′38.2″	103°05′39.4″	Landward
	T-W	5°24′45.9″	103°05′40.3″	Windward
	T-L2	5°24′42.9″	103°05′33.4″	Landward
(5) Control (C) = Batu Rakit	C	5°27′09.0″	103°02′58.4″	Control

**Notes.**

* L, Landward; W, Windward.

### Sample collection

Triplicate sediment samples for biological and environmental studies were collected from the seafloor at each of 12 substations during each monsoon season (July & December 2021) using a Ponar grab (0.023 m^2^ surface area) operated from a boat (Disco IV). A total of 36 biological samples (macrobenthos) were retrieved from the sediments of each season *via* decantation method using a 0.5 mm mesh size sieve and then preserved in 80% ethanol. The samples for sediment characteristics (grain size, total organic matter (TOM), and heavy metal) were brought back to the laboratory, in a plastic bag for further processing and analysis.

In the laboratory, macrobenthic extraction was first performed by rough sorting sediments containing macrobenthos under a stereomicroscope, followed by fine sorting to separate the organisms belonging to different taxa, and finally population quantification. The macrobenthos was identified based on the particular taxonomy group using standard references such as Day (1967) for Polychaeta, Brinkhurst (1982) for Oligochaeta, Valentich-Scott (2003) for Mollusca, Gibson & Knight-Jones (2017) for Nemertinea, Abele & Kim (1986) for Decapoda, and Amphipoda (1991) for Amphipoda. The specimens were photographed using a camera attached to an Olympus stereoscopic microscope, connected to a desktop. The specimens of different taxa were finally kept in 80% ethanol vials, labeled properly (date, locality, collector, species name), and deposited in Repository and Reference Centre (RRC) of Universiti Malaysia Terengganu for future reference.

The grain size distribution was determined by a dry sieving technique using an Octagon mechanical shaker. Sediment samples were first oven-dried (70 °C) for three days. Then, 100 g of subsamples were subjected to a dry sieve using a multilayer of sizes (4, 2, 1, 0.5, 0.25, 0.15, and 0.63 mm). Sediments retained on each layer were weighed, and data obtained were computed and analyzed in Microsoft Excel using the statistical moment method to determine the percentage of sand, silt, and clay. The sand, silt, and clay fractions were classified based on the grain diameter (mm) and mean size (phi), modified from Udden (1914) and Wentworth (1922). The textural classification was determined using the United States Department of Agriculture (USDA) textural triangle.

The sediment’s total organic matter (TOM) content was determined using the loss-on-ignition method (Bensharada et al., 2022), which calculated the weight loss after 8 h of combustion at 550 °C. The percentage of TOM was determined using the percentage loss of weight on ignition at that particular temperature.

The sediment samples for heavy metal detection were prepared using Teflon bomb digestion method (Ong et al., 2016). In the Teflon vessel, 50 mg sediments (<63 µm) were mixed with a 3:3:1 ratio of hydrofluoric, nitric, and hydrochloric acid solutions. The samples were then heated at 100^0^C for 8 h and cooled at room temperature before being diluted using Milli-Q water up to 10 ml. The concentrations of lithium (Li), aluminum (Al), chromium (Cr), lead (Pb), copper (Cu), zinc (Zn), iron (Fe), cadmium (Cd), arsenic (As), and mercury (Hg) were finally detected using an inductively coupled plasma mass spectrometry (ICP-MS).

### Data analysis

The composition of benthic assemblages at different substations was determined according to density, percentage abundance, diversity, and evenness. The diversity (Shannon–Wiener index, H’) and evenness (Pielou’s index, J’) indices were computed using Paleontological Statistics Software Package (PAST). Since the data did not meet the assumption of normal distribution (*p* < 0.05), the comparative analysis of the benthic assemblages (density, diversity, and evenness) and sediment characteristics (organic matter, heavy metal, gravel, sand, silt, and clay) at 12 different substations and two seasons were analyzed using nonparametric tests of Kruskal–Wallis and Mann–Whitney in the Statistical Package for Social Science (SPSS).

The relationship between biological parameters and sediment characteristics (organic matter, heavy metal, gravel, sand silt, and clay) was analyzed using canonical correlation analysis (CCA) from Statistical Software for Excel (XLSTAT) and principal component analysis (PCA) from SPSS. The Multiple Linear Regression (MLR) analysis was performed on the significant results to model the linear relationship between independent (sediment characteristics) and dependent (density, diversity and evenness of macrobenthos) variables. Cluster analysis was performed using the PAST Bray-Curtis similarity index to determine species composition similarity at different sampling substations, visualized through a dendrogram plot and nonmetric multidimensional scaling (NMDS) diagram. The species composition among 12 substations was evaluated using one-way analysis of similarity (ANOSIM) in PAST and the significant differences were identified based on R values ranging from 0 (groups indistinguishable) to 1 (no similarity between groups) (Clarke, 1993).

## Results

### Macrobenthos compositions

This study revealed a higher macrobenthos population during the SWM (*n* = 8,134 ind., 144 species, six phyla, 13 classes, 92 families, 124 genera) than the NEM (*n* = 3,847 ind., 123 species, five phyla, 11 classes, 80 families, 104 genera). In this regard, macrobenthic abundance differed significantly (*p* = 0.000) between the two seasons. During the SWM, station 4 (*n* = 290; 26.15%) exhibited the highest species abundance and percentage of macrobenthos, followed by station 1 (*n* = 285; 25.70%) and station 2 (*n* = 228; 20.56%) (Table 2). During the NEM, station 2 (*n* = 184; 32.6%) enumerated the highest values, followed by station 4 (*n* = 131; 23.2%) and station 5 (*n* = 112; 19.9%). Statistically, the macrobenthos abundance exhibited a significant difference (*p* = 0.018) among the five stations.

**Table 2:** The macrobenthos composition from five stations along Kuala Nerus coastal areas.

	**Average species abundance**		**Percentage abundance (%)**	
**Stations**	**SWM**	**NEM**	**SWM**	**NEM**
St. 1	285	91	25.70	16.1
St. 2	228	184	20.56	32.6
St. 3	113	46	10.19	8.2
St. 4	290	131	26.15	23.2
St. 5	193	112	17.40	19.9

In line with a higher number of individuals recorded, six phyla of macrobenthos were recorded during the SWM, whereas five phyla during the NEM (Table 3). They shared four common phyla for both seasons, namely Mollusca, Annelida, Arthropoda, and Echinodermata, with other phyla of Chordata and Nematoda found during SWM and Nemertea during NEM. Overall, for both seasons, the highest species composition was represented by phylum Mollusca with 60%–99.3% (SWM: *n* = 82 species, *n* = 2308 ind.; NEM: *n* = 92 species, *n* = 1,219 ind.), followed by Annelida with 0.5%–32.6% (SWM: *n* = 44 species, *n* = 282 ind.; NEM: *n* = 18 species, *n* = 26 ind.) and Arthropoda with less than 1% (SWM: *n* = 12 species, *n* = 68 ind.; NEM: *n* = 9 species, *n* = 32 ind.) (Table 3).

**Table 3:** Distribution and abundance of macrobenthos (Phyla level) throughout 12 sampling points in coastal defence structure areas in Kuala Nerus, Terengganu.

	**Percentage abundance (%)**																							
	**St. 1**						**St. 2**				**St. 3**						**St. 4**						**St. 5**	
**Stations**	**G-L1**		**G-W**		**G-L2**		**S-L**		**S-W**		**B-L1**		**B-W**		**B-L2**		**T-L1**		**T-W**		**T-L2**		**BR**	
**Phyla/seasons**	**SWM**	**NEM**	**SWM**	**NEM**	**SWM**	**NEM**	**SWM**	**NEM**	**SWM**	**NEM**	**SWM**	**NEM**	**SWM**	**NEM**	**SWM**	**NEM**	**SWM**	**NEM**	**SWM**	**NEM**	**SWM**	**NEM**	**SWM**	**NEM**
Mollusca	89.25	86.21	84.27	98.66	60.08	92.57	90.49	89.01	78.72	99.28	69.45	98.10	81.17	95.62	87.22	96.58	97.65	92.92	79.26	96.89	91.37	88.36	92.55	94.76
Annelida	9.26	9.43	11.12	1.34	32.67	0.00	6.69	4.20	6.88	0.41	18.54	1.57	14.70	3.75	8.39	1.33	2.30	2.17	10.54	2.06	7.29	4.14	6.20	1.61
Arthropoda	0.81	1.49	1.73	0.00	2.99	5.59	1.66	6.79	2.97	0.22	11.00	0.33	1.55	0.63	3.49	1.39	0.06	4.91	8.12	1.05	0.90	6.84	1.12	3.63
Echinodermata	0.67	2.88	2.30	0.00	3.89	1.84	0.85	0.00	11.00	0.00	1.01	0.00	2.19	0.00	0.41	0.69	0.00	0.00	1.39	0.00	0.44	0.66	0.13	0.00
Chordata	0.00	0.00	0.14	0.00	0.23	0.00	0.17	0.00	0.13	0.00	0.00	0.00	0.39	0.00	0.48	0.00	0.00	0.00	0.00	0.00	0.00	0.00	0.00	0.00
Nematoda	0.00	0.00	0.44	0.00	0.14	0.00	0.14	0.00	0.29	0.00	0.00	0.00	0.00	0.00	0.00	0.00	0.00	0.00	0.69	0.00	0.00	0.00	0.00	0.00
Nemertea	0.00	0.00	0.00	0.00	0.00	0.00	0.00	0.00	0.00	0.08	0.00	0.00	0.00	0.00	0.00	0.00	0.00	0.00	0.00	0.00	0.00	0.00	0.00	0.00
**Average density (no. of ind./m^**2**^)**																								
Mollusca	14,623.188	1,362.319	7,710.145	8,942.029	7,057.971	1,130.435	9,028.986	19,85.507	7,971.014	13,652.174	4,826.087	2,971.014	3,304.348	1,695.652	3,362.319	1,057.971	13,043.478	4,057.971	6,724.638	1,913.043	14,840.580	9,550.725	7,855.072	4,695.652
Annelida	1,652.174	130.435	942.029	115.942	3,753.623	0.000	637.681	101.449	695.652	57.971	1,159.420	43.478	536.232	86.957	347.826	14.493	173.913	86.957	869.565	57.971	1,057.971	405.797	434.783	43.478
Arthropoda	130.435	21.739	144.928	0.000	304.348	57.971	173.913	159.420	289.855	28.986	739.130	14.493	57.971	14.493	159.420	28.986	14.493	202.899	695.652	28.986	144.928	695.652	86.957	144.928
Echinodermata	57.971	57.971	202.899	0.000	521.739	28.986	86.957	0.000	869.565	0.000	72.464	0.000	86.957	0.000	14.493	14.493	0.000	0.000	115.942	0.000	57.971	72.464	14.493	0.000
Chordata	0.000	0.000	14.493	0.000	28.986	0.000	14.493	0.000	14.493	0.000	0.000	0.000	14.493	0.000	14.493	0.000	0.000	0.000	0.000	0.000	0.000	0.000	0.000	0.000
Nematoda	0.000	0.000	28.986	0.000	14.493	0.000	14.493	0.000	14.493	0.000	0.000	0.000	0.000	0.000	0.000	0.000	0.000	0.000	57.971	0.000	0.000	0.000	0.000	0.000
Nemertea	0.000	0.000	0.000	0.000	0.000	0.000	0.000	0.000	0.000	14.493	0.000	0.000	0.000	0.000	0.000	0.000	0.000	0.000	0.000	0.000	0.000	0.000	0.000	0.000
Total	16,463.768	1,572.464	9,043.48	9,057.971	11,681.16	1,217.392	9,956.523	2,246.376	9,855.072	13,753.624	6,797.101	3,028.985	4,000.001	1,797.102	3,898.551	1,115.943	13,231.884	4,347.827	8,463.768	2,000	16,101.45	10,724.638	8,391.305	4,884.058

**Notes.**

* G, Groyne; S, Semi-enclosed jetty-type breakwater; B, Parallel breakwater without tombolo; T, Parallel breakwater with tombolo; BR, Batu Rakit, L, Landward; W, Windward.

**Figure 2:**
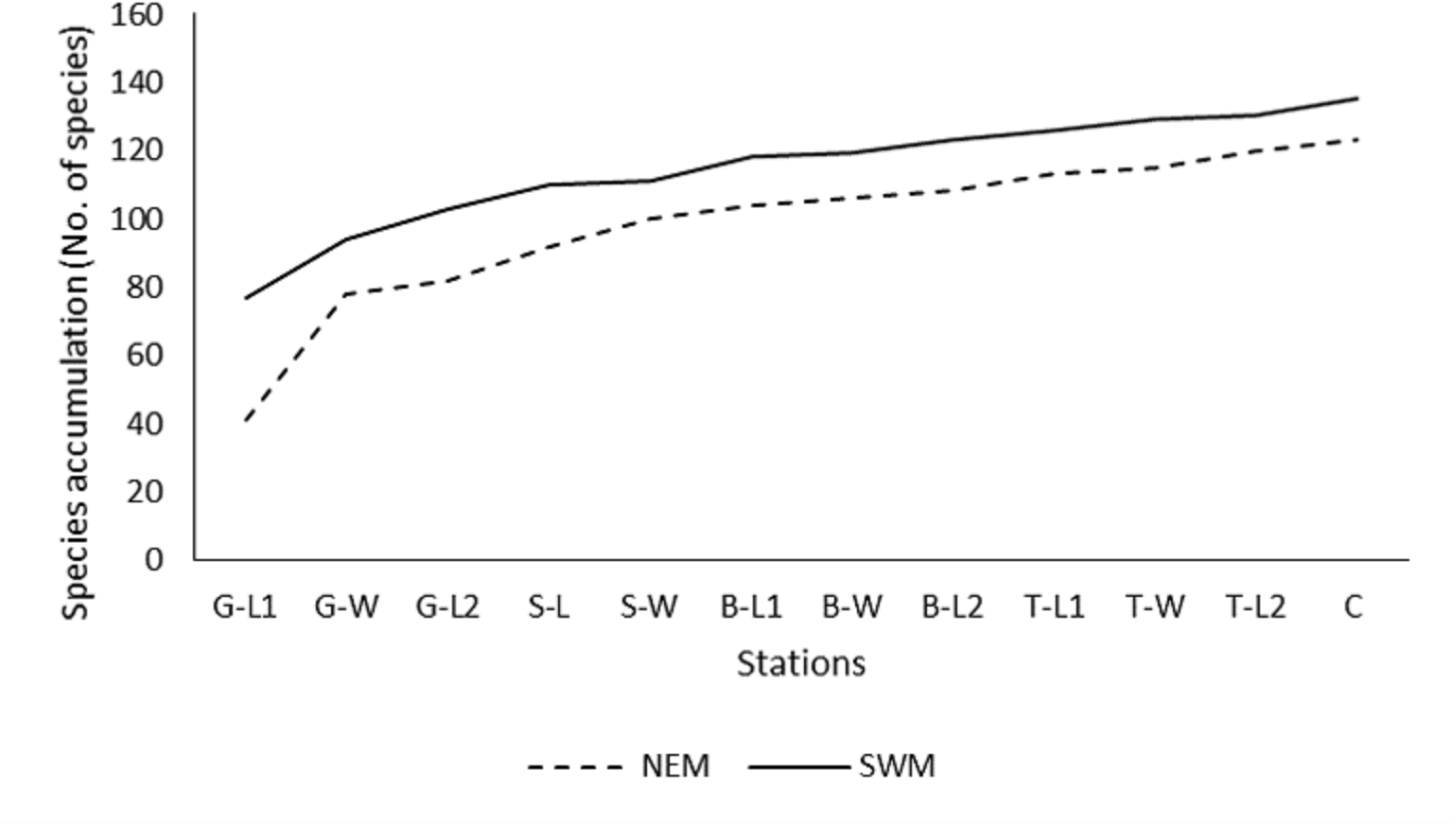
Species accumulation curve (SAC). Species accumulation curve (SAC) of macrobenthos in SWM and NEM in Kuala Nerus coastal defence structure area.

Throughout the substations, starting from G-L1 in both seasons, the species accumulation curve (SAC) increases rapidly toward the next substations of G-W (Fig. 2). However, as the sampling progressed to other substations (G-W and above), the SAC gradually increased toward the end. It stopped at approximately 135 (SWM) and 125 (NEM) species. Overall, the SAC demonstrated that the gentle slope line in both seasons yielded fewer new species, particularly during the SWM because most species were found earlier throughout the sampling period and stations. The slope line is expected to level off as sampling activities continue to further stations, resulting in fewer new species or records.

At substation G-W, a drastic decreasing pattern of Annelida was observed between those two seasons (SWM: 11.1%; 942.029 ind./m^2^ to NEM: 1.34%; 115.942 ind./m^2^) (Table 3). During the NEM, only two phyla were found at the station, namely the Mollusca and Annelida, whereas during the SWM, all phyla were present, except Nemertea. Annelida in G-L2 during the SWM showed a greater difference (*n* = 86 ind., 32.6%; 3,753.623 ind./m^2^) than the other substations, which recorded less than 30 individuals, 20% per substation, and 2,000 ind./m^2^ (Table 3).

Spatially, all substations in station 1 exhibited a similar population size of macrobenthos between NEM and SWM, in contrast to the other substations, which exhibited a pattern of smaller population size during NEM and higher during SWM (Tables 3 and 4). The lowest average abundance of macrobenthos in station 3 (*n* = 113-SWM and 46-NEM; range from 1,115.943 to 6,797.101 ind./m^2^) is almost one-third of the average abundance of individuals in station 1 (n = 285-SWM and 91-NEM; range from 1,217.392 to 11,681.16 ind./m^2^) and 4 (*n* = 290-SWM and 131-NEM; range from 2,000 to 16,101.45 ind./m^2^) with the same number of substations (Tables 2 and 3). In addition, S-W exhibited an opposite pattern of the population size of macrobenthos with a higher density recorded during the NEM (13,753.624 ind./m^2^) compared with the SWM (9,855.072 ind./m^2^) (Tables 3 and 4). Statistically, macrobenthos density was significantly different between those two seasons (*p* = 0.000), five stations (*p* = 0.000), and 12 substations (*p* = 0.006).

**Table 4:** Distribution and abundance of macrobenthos (Classes level) throughout 12 sampling points in coastal defence structure areas in Kuala Nerus, Terengganu.

	**Percentage abundance (%)**																							
	**St. 1**						**St. 2**				**St. 3**						**St. 4**						**St. 5**	
**Stations**	**G-L1**		**G-W**		**G-L2**		**S-L**		**S-W**		**B-L1**		**B-W**		**B-L2**		**T-L1**		**T-W**		**T-L2**		**BR**	
**Classes/ seasons**	**SWM**	**NEM**	**SWM**	**NEM**	**SWM**	**NEM**	**SWM**	**NEM**	**SWM**	**NEM**	**SWM**	**NEM**	**SWM**	**NEM**	**SWM**	**NEM**	**SWM**	**NEM**	**SWM**	**NEM**	**SWM**	**NEM**	**SWM**	**NEM**
Gastropoda	70.00	64.47	59.60	72.86	41.96	26.26	58.04	54.00	44.18	76.44	16.43	91.57	38.65	74.03	40.87	53.50	81.11	60.67	42.34	42.28	80.74	74.20	64.47	74.60
Bivalvia	18.75	21.73	24.67	24.74	17.98	61.32	31.33	34.54	34.55	22.51	52.53	5.95	41.63	21.59	46.36	42.39	16.40	31.20	36.92	54.60	10.31	7.24	27.91	18.81
Scaphopoda	0.51	0.00	0.00	1.06	0.14	4.99	1.12	0.47	0.00	0.34	0.48	0.57	0.89	0.00	0.00	0.69	0.14	1.05	0.00	0.00	0.32	6.93	0.17	1.35
Polychaeta	8.99	9.43	11.12	1.34	32.67	0.00	6.56	4.20	6.88	0.41	17.90	1.57	14.70	3.75	8.39	1.33	2.30	2.17	10.54	2.06	7.29	4.14	6.20	1.61
Clitellata	0.27	0.00	0.00	0.00	0.00	0.00	0.14	0.00	0.00	0.00	0.64	0.00	0.00	0.00	0.00	0.00	0.00	0.00	0.00	0.00	0.00	0.00	0.00	0.00
Malacostraca	0.68	1.49	1.73	0.00	2.72	4.74	1.53	6.79	2.97	0.00	10.41	0.33	1.16	0.00	3.49	1.39	0.06	4.91	7.78	1.05	0.90	6.84	0.87	3.63
Ostracoda	0.13	0.00	0.00	0.00	0.27	0.85	0.00	0.00	0.00	0.00	0.58	0.00	0.00	0.00	0.00	0.00	0.00	0.00	0.18	0.00	0.00	0.00	0.25	0.00
Hexanauplia	0.00	0.00	0.00	0.00	0.00	0.00	0.13	0.00	0.00	0.22	0.00	0.00	0.39	0.63	0.00	0.00	0.00	0.00	0.16	0.00	0.00	0.00	0.00	0.00
Echinoidea	0.61	2.88	1.86	0.00	3.51	0.00	0.27	0.00	11.00	0.00	0.64	0.00	2.19	0.00	0.41	0.00	0.00	0.00	1.04	0.00	0.33	0.35	0.00	0.00
Holothuroidea	0.00	0.00	0.00	0.00	0.00	0.00	0.00	0.00	0.00	0.00	0.16	0.00	0.00	0.00	0.00	0.00	0.00	0.00	0.00	0.00	0.00	0.00	0.00	0.00
Ophiuroidea	0.06	0.00	0.44	0.00	0.37	1.84	0.58	0.00	0.00	0.00	0.21	0.00	0.00	0.00	0.00	0.00	0.00	0.00	0.35	0.00	0.11	0.30	0.13	0.00
Leptocardii	0.00	0.00	0.14	0.00	0.23	0.00	0.17	0.00	0.13	0.00	0.00	0.00	0.39	0.00	0.48	0.00	0.00	0.00	0.00	0.00	0.00	0.00	0.00	0.00
Nematoda	0.00	0.00	0.44	0.00	0.14	0.00	0.14	0.00	0.29	0.00	0.00	0.00	0.00	0.00	0.00	0.00	0.00	0.00	0.69	0.00	0.00	0.00	0.00	0.00
Asteroidea	0.00	0.00	0.00	0.00	0.00	0.00	0.00	0.00	0.00	0.00	0.00	0.00	0.00	0.00	0.00	0.69	0.00	0.00	0.00	0.00	0.00	0.00	0.00	0.00
Nemertea	0.00	0.00	0.00	0.00	0.00	0.00	0.00	0.00	0.00	0.08	0.00	0.00	0.00	0.00	0.00	0.00	0.00	0.00	0.00	0.00	0.00	0.00	0.00	0.00
	**Average Density (No. of ind./m^**2**^)**																							
Gastropoda	11,231.88	1,000.00	5,536.23	6,565.22	5,043.48	391.30	5,826.09	1,231.88	4,376.81	10,478.26	1,115.94	2,782.61	1,565.22	1,318.84	1,623.19	637.68	11,463.77	2,782.61	3,521.74	826.09	13,144.93	8,115.94	5,884.06	3,811.59
Bivalvia	3,275.36	362.32	2,173.91	2,275.36	2,000.00	695.65	3,101.45	739.13	3,594.20	3,130.44	3,681.16	173.91	1,710.15	376.81	1,739.13	405.80	1,565.22	1,217.39	3,202.90	1,086.96	1,637.68	811.59	1,956.52	840.58
Scaphopoda	115.94	0.00	0.00	101.45	14.49	43.48	101.45	14.49	0.00	43.48	28.99	14.49	28.99	0.00	0.00	14.49	14.49	57.97	0.00	0.00	57.97	623.19	14.49	43.48
Polychaeta	1,637.68	130.44	942.03	115.94	3,753.62	0.00	623.19	101.45	695.65	57.97	1,101.45	43.48	536.23	86.96	347.83	14.49	173.91	86.96	869.57	57.97	1,057.97	405.80	434.78	43.48
Clitellata	14.49	0.00	0.00	0.00	0.00	0.00	14.49	0.00	0.00	0.00	57.97	0.00	0.00	0.00	0.00	0.00	0.00	0.00	0.00	0.00	0.00	0.00	0.00	0.00
Malacostraca	101.45	21.74	144.93	0.00	275.36	43.48	159.42	159.42	289.86	0.00	695.65	14.49	43.48	0.00	159.42	28.99	14.49	202.90	666.67	28.99	144.93	695.65	57.97	144.93
Ostracoda	28.99	0.00	0.00	0.00	28.99	14.49	0.00	0.00	0.00	0.00	43.48	0.00	0.00	0.00	0.00	0.00	0.00	0.00	14.49	0.00	0.00	0.00	28.99	0.00
Hexanauplia	0.00	0.00	0.00	0.00	0.00	0.00	14.49	0.00	0.00	28.99	0.00	0.00	14.49	14.49	0.00	0.00	0.00	0.00	14.49	0.00	0.00	0.00	0.00	0.00
Echinoidea	43.48	57.97	159.42	0.00	478.26	0.00	28.99	0.00	869.57	0.00	43.48	0.00	86.96	0.00	14.49	0.00	0.00	0.00	86.96	0.00	43.48	28.99	0.00	0.00
Holothuroidea	0.00	0.00	0.00	0.00	0.00	0.00	0.00	0.00	0.00	0.00	14.49	0.00	0.00	0.00	0.00	0.00	0.00	0.00	0.00	0.00	0.00	0.00	0.00	0.00
Ophiuroidea	14.49	0.00	43.48	0.00	43.48	28.99	57.97	0.00	0.00	0.00	14.49	0.00	0.00	0.00	0.00	0.00	0.00	0.00	28.99	0.00	14.49	43.48	14.49	0.00
Leptocardii	0.00	0.00	14.49	0.00	28.99	0.00	14.49	0.00	14.49	0.00	0.00	0.00	14.49	0.00	14.49	0.00	0.00	0.00	0.00	0.00	0.00	0.00	0.00	0.00
Nematoda	0.00	0.00	28.99	0.00	14.49	0.00	14.49	0.00	14.49	0.00	0.00	0.00	0.00	0.00	0.00	0.00	0.00	0.00	57.97	0.00	0.00	0.00	0.00	0.00
Asteroidea	0.00	0.00	0.00	0.00	0.00	0.00	0.00	0.00	0.00	0.00	0.00	0.00	0.00	0.00	0.00	14.49	0.00	0.00	0.00	0.00	0.00	0.00	0.00	0.00
Nemertea	0.00	0.00	0.00	0.00	0.00	0.00	0.00	0.00	0.00	14.49	0.00	0.00	0.00	0.00	0.00	0.00	0.00	0.00	0.00	0.00	0.00	0.00	0.00	0.00
Total	16,463.77	1,572.46	9,043.48	9,057.97	11,681.16	1,217.39	9,956.52	2,246.38	9,855.07	13,753.62	6,797.10	3,028.99	4,000.00	1,797.10	3,898.55	1,115.94	13,231.88	4,347.83	8,463.77	2,000.00	16,101.45	10,724.64	8,391.31	4,884.06

**Notes.**

* G, Groyne; S, Semi-enclosed jetty-type breakwater; B, Parallel breakwater without tombolo; T, Parallel breakwater with tombolo; BR, Batu Rakit, L, Landward; W, Windward.

During both seasons, 15 classes of macrobenthos were found in the taxonomic rank of class level, with 13 classes recorded during the SWM and 11 classes during the NEM (Table 4). They shared nine common classes for both seasons, namely the Gastropoda, Bivalvia, Scaphopoda, Polychaeta, Malacostraca, Ostracoda, Hexanauplia, Echinoidea, and Ophiuroidea. Overall, the Gastropoda had the highest species composition with 16.4%–91.6% (SWM: *n* = 66 species, 1618 ind.; NEM: *n* = 78 species, 919 ind.), followed by Bivalvia with 5.95%–61.3% (SWM: *n* = 13 species, 682 ind.; NEM: *n* = 12 species, 279 ind.) and Polychaeta with 0%–17.9% (SWM: *n* = 43 species, 280 ind.; NEM: *n* = 18 species, 26 ind.) (Table 4).

Despite having the highest percentage and density of Annelida (Polychaeta) during the SWM (32.6%; 3,753 ind./m^2^), substation G-L2 had zero Annelida (Polychaeta) during the NEM season (Tables 3 and 4). The percentage was highly substituted by Bivalvia (61.32%; 695.652 ind./m^2^ in NEM), with only 17.98% and 2,000 ind./m^2^ during SWM. In B-L1, Gastropoda and Bivalvia exhibited a reverse pattern between those two seasons, with the lowest Gastropoda recorded during the SWM (16.43%; 1,115.942 ind./m^2^) being replaced by the highest Gastropoda recorded during the NEM season (91.57%; 2,782.609 ind./m^2^). The highest Bivalvia documented during the SWM (52.52%; 3,681.159 ind./m^2^) was replaced by the lowest Bivalvia documented during the NEM (5.95%; 173.913 ind./m^2^).

The highest density of organisms recorded was *Finella* sp. from phylum Mollusca and Class Gastropoda (37,652 ind./m^2^), followed by *Nucula* sp. (Mollusca, Bivalvia; 32,434.78 ind./m^2^) and *Umbonium* sp. (Mollusca, Gastropoda; 31,130.43 ind./m^2^) across all stations during the SWM. During the NEM season, they were subsequently replaced by Gastropoda of *Pirenella cingulata,* which had the highest density of 17,130.4 ind./m^2^, followed by *Alvania* sp. (16,913 ind./m^2^) and *Scaliola* sp. (12,434.8 ind./m^2^).

**Figure 3:**
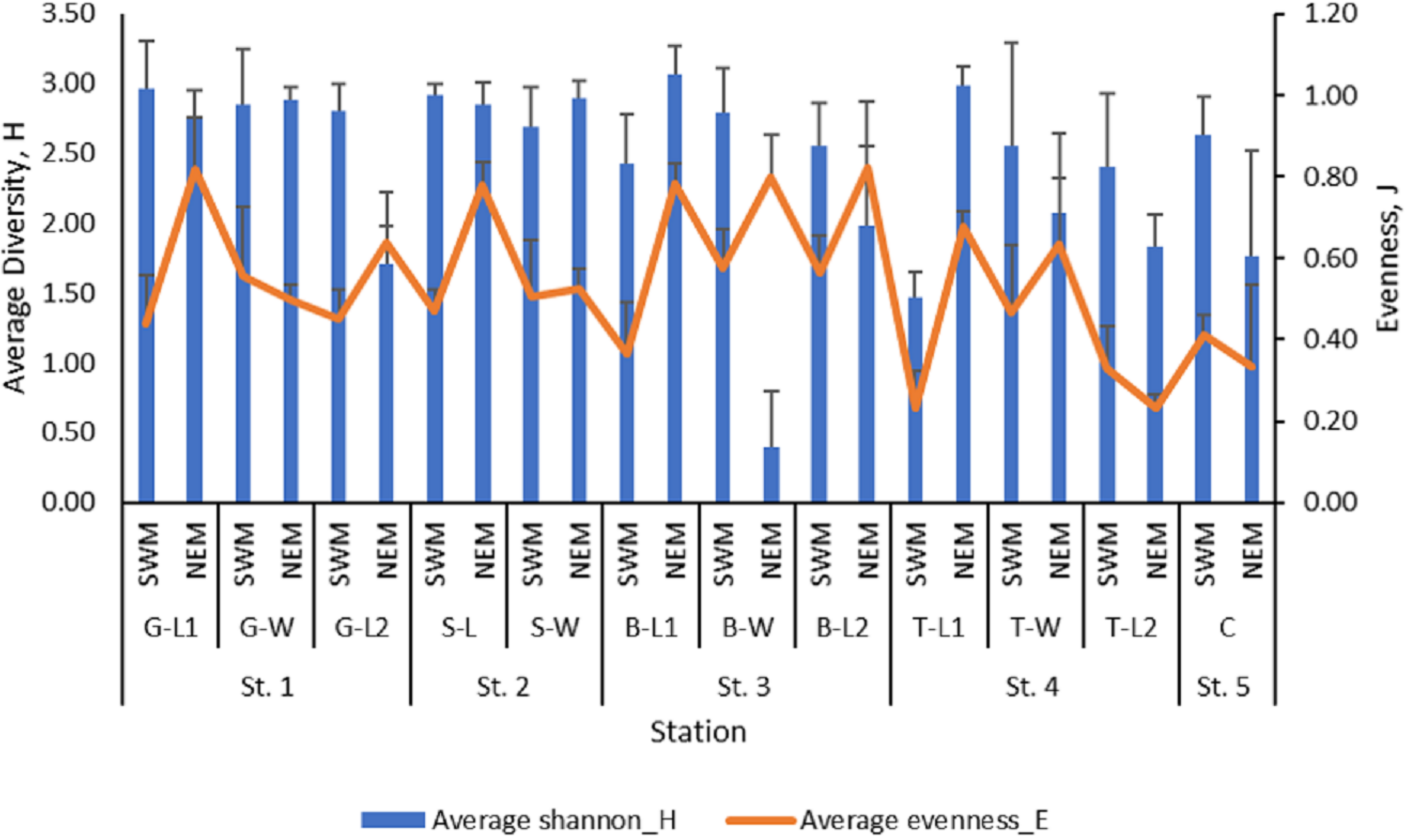
The macrobenthos diversity (H’) and evenness (J’) at each sub-station along Kuala Nerus coastal area, in relation to coastal defence structure and two main seasons (SWM & NEM).

The mean value of macrobenthos diversity across all substations ranged from 1.47 (T-L1) to 2.96 (G-L1) during the SWM (Fig. 3), while during the NEM, the diversity value ranged from 1.71 (G-L2) to 3.07 (B-L1). The SWM recorded values ranging from 0.23 (T-L1) to 0.58 (B-W) for the evenness index, while the NEM recorded values ranging from 0.23 (T-L2) to 0.82 (B-L2). There was a significant difference in the diversity (*p* = 0.010) and evenness ( *p* = 0.010) indices between those two seasons. However, no significant difference was observed between the five stations (p = H’: 0.477; J’: 0.470).

### The similarity of species composition

**Figure 4:**
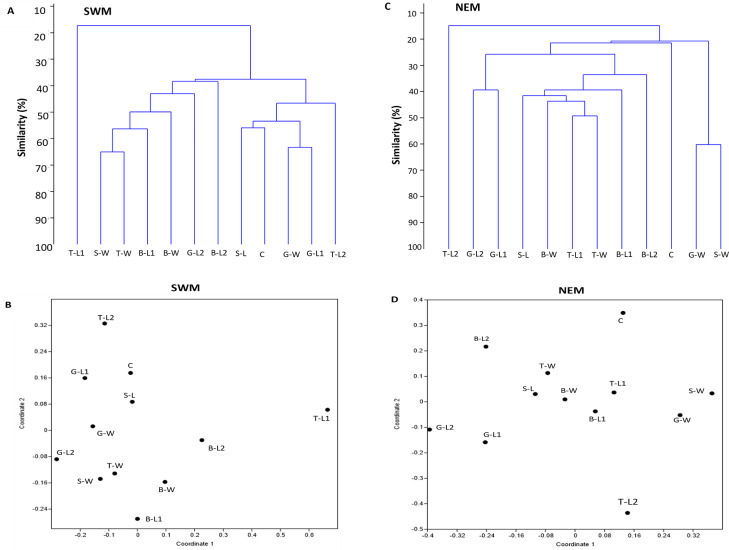
Bray–Curtis similarity based on hierarchical clustering of substations during SWM (A and B) and NEM (C and D), showing the macrobenthic assemblage pattern in Kuala Nerus coastal defence structure. The similarity manifested through dendrogram (A, C) and nonmetric multidimensional scaling, NMDS (B, D).

For each season, two separate clusters were formed, separating substation T-L1 from all other substations (SWM) and T-L2 from the rest of the substations (NEM), at 18% and 15% similarity levels, respectively (Figs. 4A, 4C). Following the extreme dissimilarity, the next line that merged revealed three separate clusters of species composition, in which, during the SWM, at approximately 38% similarity level, Cluster 1 consisted of T-L1 only; Cluster 2 consisted of S-W, T-W, B-L2, B-W, G-L2, and B-L2; and Cluster 3 consists of S-L, C, G-W, G-L1 and T-L2. At approximately 22% similarity level, Cluster 1 in NEM comprises only T-L2; Cluster 2 comprises G-L2, G-L1, S-L, B-W, T-L1, T-W B-L1, B-L2, and C; and Cluster 3 comprises G-W and S-W. At a higher stage of hierarchy (50% similarity level), more clusters of substations formed during both seasons, with SWM recording fewer total clusters (seven) than NEM (11). All substations eventually become separate clusters (12 clusters each season) at 66% (SWM) and 60% similarity levels. During the SWM, S-W and T-W, S-L and C, and G-W and G-L1 substations, they shared the closest species composition, as indicated by the merged lines at approximately 76% similarity level. During the NEM, G-L2 and G-L1, T-L1 and T-W, and G-W and S-W substations were grouped separately at a 62% similarity level and shared the closest species composition. Similarly, the NMDS analysis confirmed the dendrogram output (Figs. 4B, 4D). The ANOSIM test shows that the species composition of macrobenthos in different substations has a global R-value of 1, indicating the dissimilarities between the stations.

### Sediment characteristics

**Figure 5:**
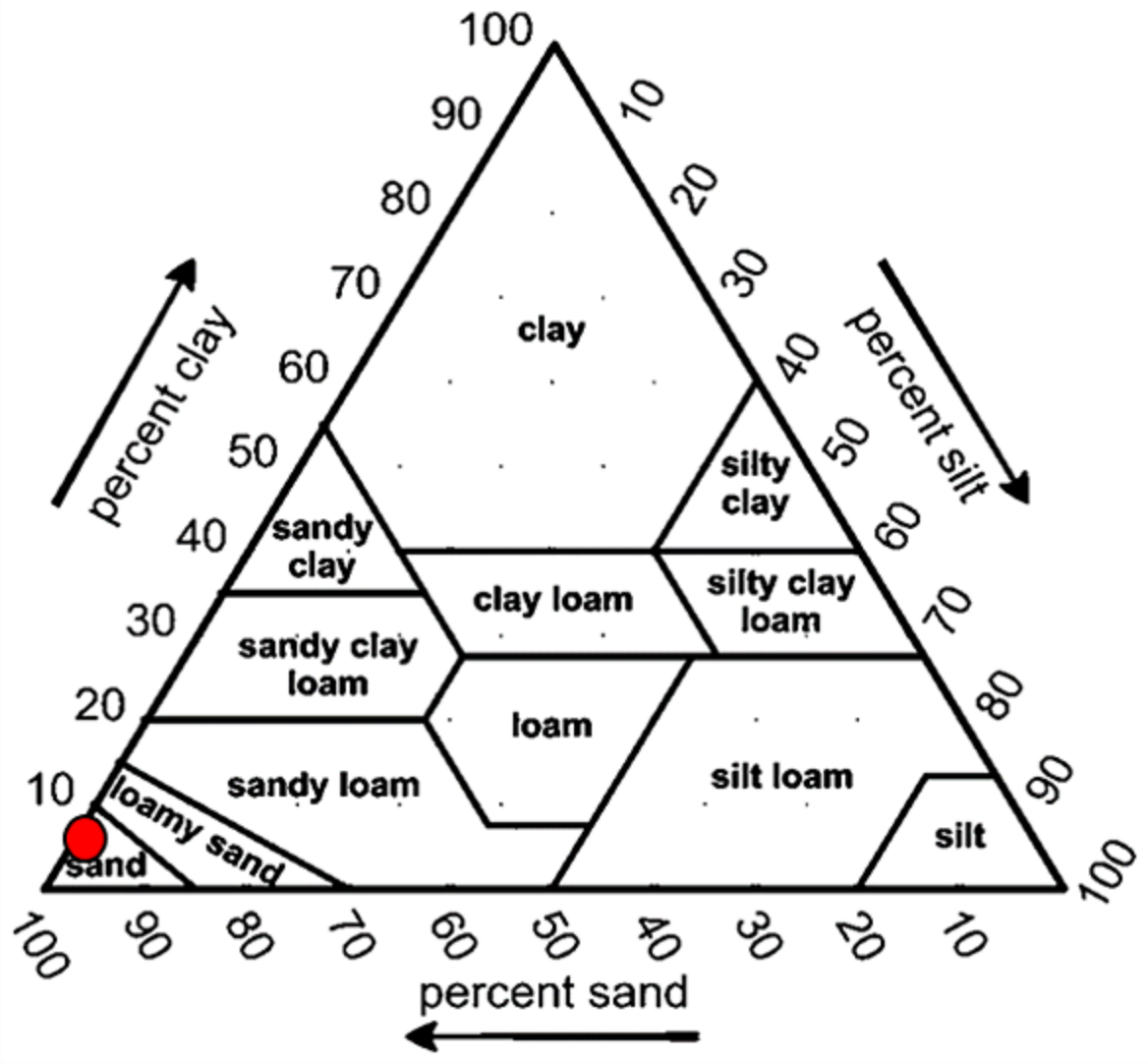
Soil classifications at coastal defence structure areas in Kuala Nerus, Terengganu, plotted on the USDA texture Triangle.

**Figure 6:**
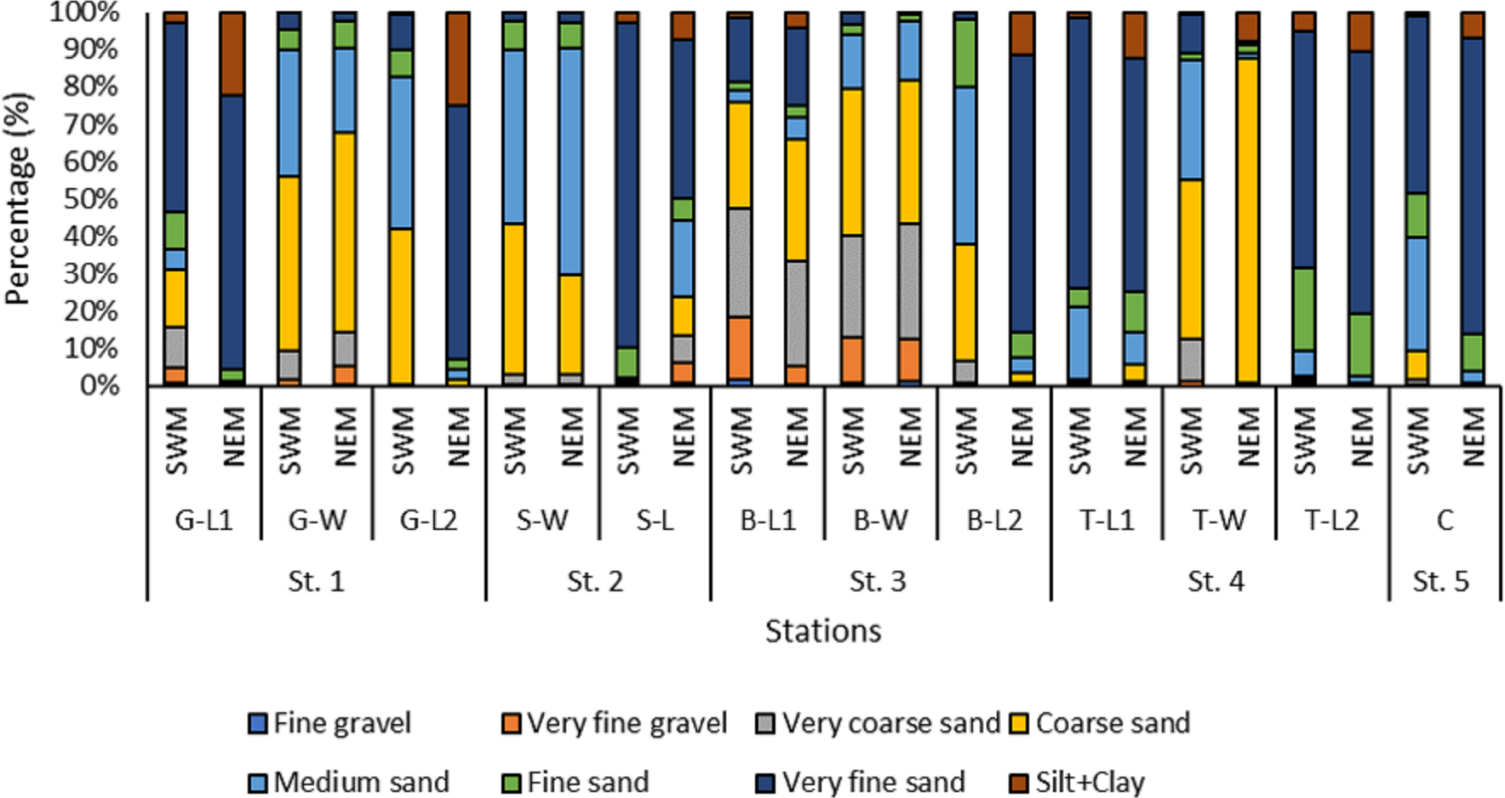
The grain size distribution across substations along Kuala Nerus coastal area, during southwest and northeast monsoon seasons.

Overall, the surface sediments at the coastal defense structure areas of Kuala Nerus, Terengganu are mostly governed by sand (up to 95%) with a composition of very fine, coarse, and medium sands. The textural classification of the sand throughout the substations and seasons is shown in the USDA soil texture triangle (Fig. 5). Most stations were dominated by the same type of sediments for both seasons, either very fine, coarse, or medium sands, except for sheltered stations such as G-L2, B-L1, and B-L2, which underwent sediment downgrading from dry to wet seasons (Fig. 6). For example, G-L2 recorded coarse sand (41.62%) during the SWM and very fine sand (67.68%) during the NEM. Observations during the sampling in both seasons verified that most “landward” stations were dominated by very fine sand (44.26%–86.70%), particularly in G-L1, S-L, T-L1, T-L2, and BR. In contrast, “windward” stations such as G-W, G-L2, B-W, and T-W were dominated by coarse sand (38.56%–86.84%). The medium sand was recorded at S-W for both dry and wet seasons, with 46.79% and 60.47%, respectively.

**Figure 7:**
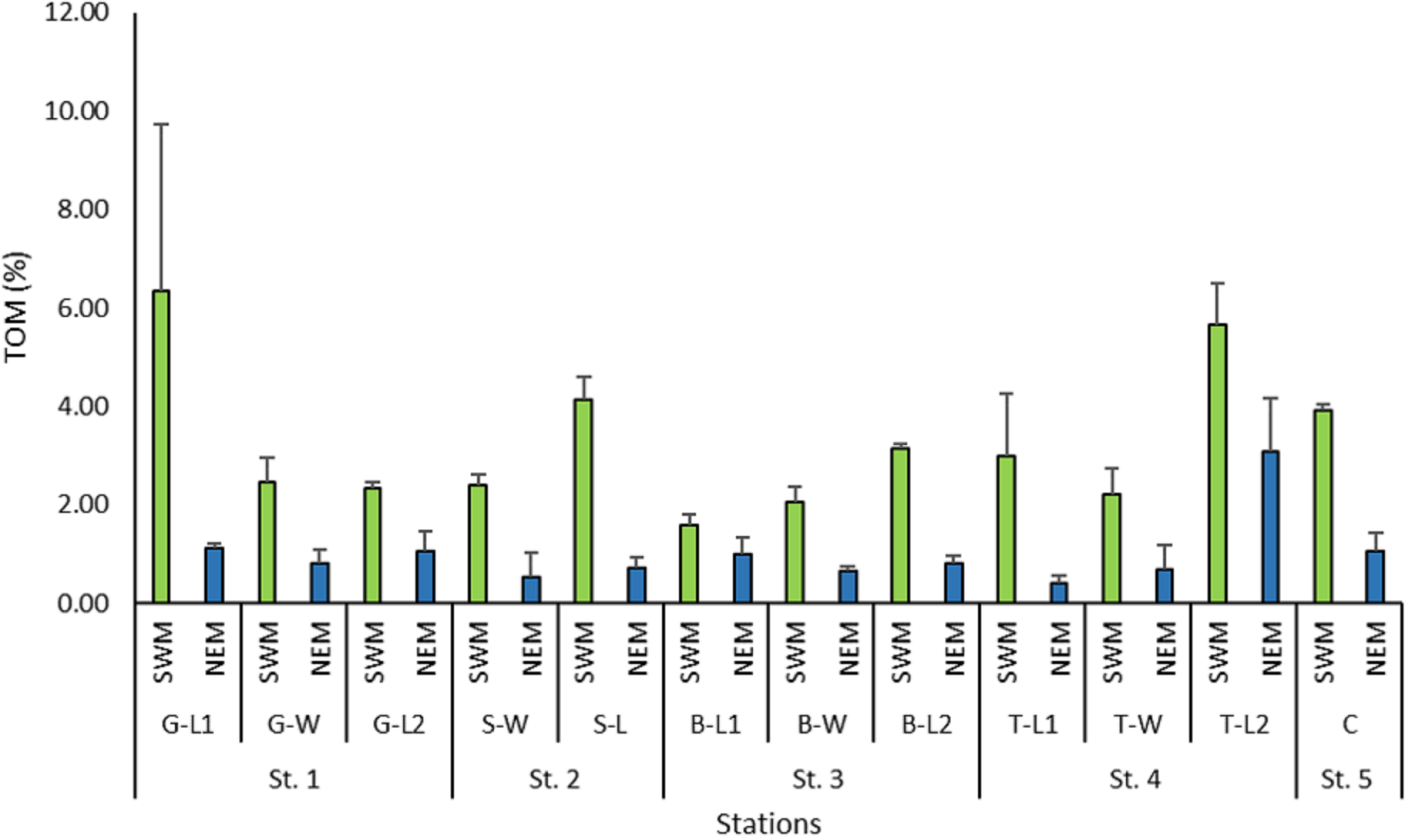
Percentage of total organic matter (%) across substations along Kuala Nerus coastal area, in relation to coastal defence structure, based on monsoonal seasons.

The total organic matter (TOM) recorded across the stations during the NEM season was reasonably lower (0.4%–3.1%) than that of the SWM season (1.6%–6.33%) (Fig. 7). The TOM percentage at the 12 substations along the Kuala Nerus coastal area did not show any significant difference (*p* = 0.853), but it did differ significantly between seasons (*p* = 0.000).

The metal concentrations of Li, Al, Cr, Cu, Zn, Cd, Pb, As, and Hg in the surface sediments collected at the 12 substations were within the range of 0.06–6.81, 0.83–3.55, 0.01–18.78, 0.14–10.59, 0.09–7.68, 0.01–0.07, 0.01–2.16, 0.01–1.50, and 0.001–0.003 ppm, respectively, for both seasons (Table 5). No significant correlation between the various heavy metals and the stations (*p* = 0.083–0.800) was observed. Temporally, a significant correlation was observed between Al and As (*p* = 0.018 and 0.018). The concentrations are within the permissible range for the upper continental crust (UCC) (Table 5), as the values are considerably lower than the specified limits.

**Table 5:** Heavy metal concentrations (ppm) at the surface sediment of 12 points in two Asian monsoon systems, southwest and northeast monsoon seasons in Kuala Nerus, Terengganu, Malaysia.

	St. 1						St. 2				St. 3						St. 4						St. 5		
Stations	**G-L1**		**G-W**		**G-L2**		**S-W**		**S-W**		**BL-1**		**B-W**		**B-L2**		**T-L1**		**T-W**		**T-L2**		**C**		
Metal/ seasons	**SWM**	**NEM**	**SWM**	**NEM**	**SWM**	**NEM**	**SWM**	**NEM**	**SWM**	**NEM**	**SWM**	**NEM**	**SWM**	**NEM**	**SWM**	**NEM**	**SWM**	**NEM**	**SWM**	**NEM**	**SWM**	**NEM**	**SWM**	**NEM**	**UCC**
Li	4.93	4.71	1.94	3.17	3.16	5.78	2.05	2.63	5.08	0.06	2.08	3.57	1.92	4.67	3.81	3.08	4.61	4.02	5.21	4.78	6.62	6.81	4.64	5.08	22.00
AI	3.20	3.05	3.52	3.10	3.20	3.17	3.39	3.31	3.15	0.83	3.55	3.40	3.46	3.40	3.50	3.32	3.44	3.07	3.39	3.08	3.36	3.27	3.11	3.20	77440.00
Cr	0.88	0.84	1.54	0.86	1.59	0.88	1.37	19.44	1.79	0.01	2.20	0.94	0.90	0.96	0.97	8.44	6.78	2.14	0.94	0.85	5.41	1.77	15.31	18.78	35.00
Cu	1.98	3.48	0.95	0.88	0.93	4.01	1.03	0.19	0.54	1.43	0.82	0.37	1.23	0.78	10.59	0.42	0.14	0.44	2.35	5.17	0.62	0.46	0.35	0.45	14.30
Zn	7.68	6.57	3.44	3.11	3.48	5.62	2.50	3.15	5.92	0.09	4.54	3.88	2.01	3.64	6.74	4.29	6.28	6.51	7.46	7.54	5.68	7.24	4.10	6.55	52.00
Cd	0.04	0.02	0.00	0.01	0.01	0.02	0.00	0.01	0.02	0.00	0.06	0.00	0.00	0.00	0.03	0.02	0.02	0.04	0.03	0.07	0.02	0.01	0.01	0.02	0.10
Pb	1.66	1.15	0.72	0.39	0.66	1.46	0.56	0.54	1.35	0.00	0.86	0.37	0.34	0.40	1.22	0.73	1.19	1.01	1.57	1.57	2.16	1.84	1.23	1.28	16.00
As	1.23	0.80	1.42	0.42	0.50	0.82	0.97	0.41	1.50	0.01	0.96	0.29	0.41	0.98	1.17	0.45	0.74	0.64	1.33	0.96	1.41	1.40	1.07	0.77	2.00
Hg	0.00	0.00	0.00	0.00	0.00	0.00	0.00	0.00	0.00	0.00	0.00	0.00	0.00	0.00	0.00	0.00	0.00	0.00	0.00	0.00	0.00	0.00	0.00	0.00	0.06

**Notes.**

UCC, Upper continental Crust value.

### Relationship between biological and sediment characteristics

**Figure 8:**
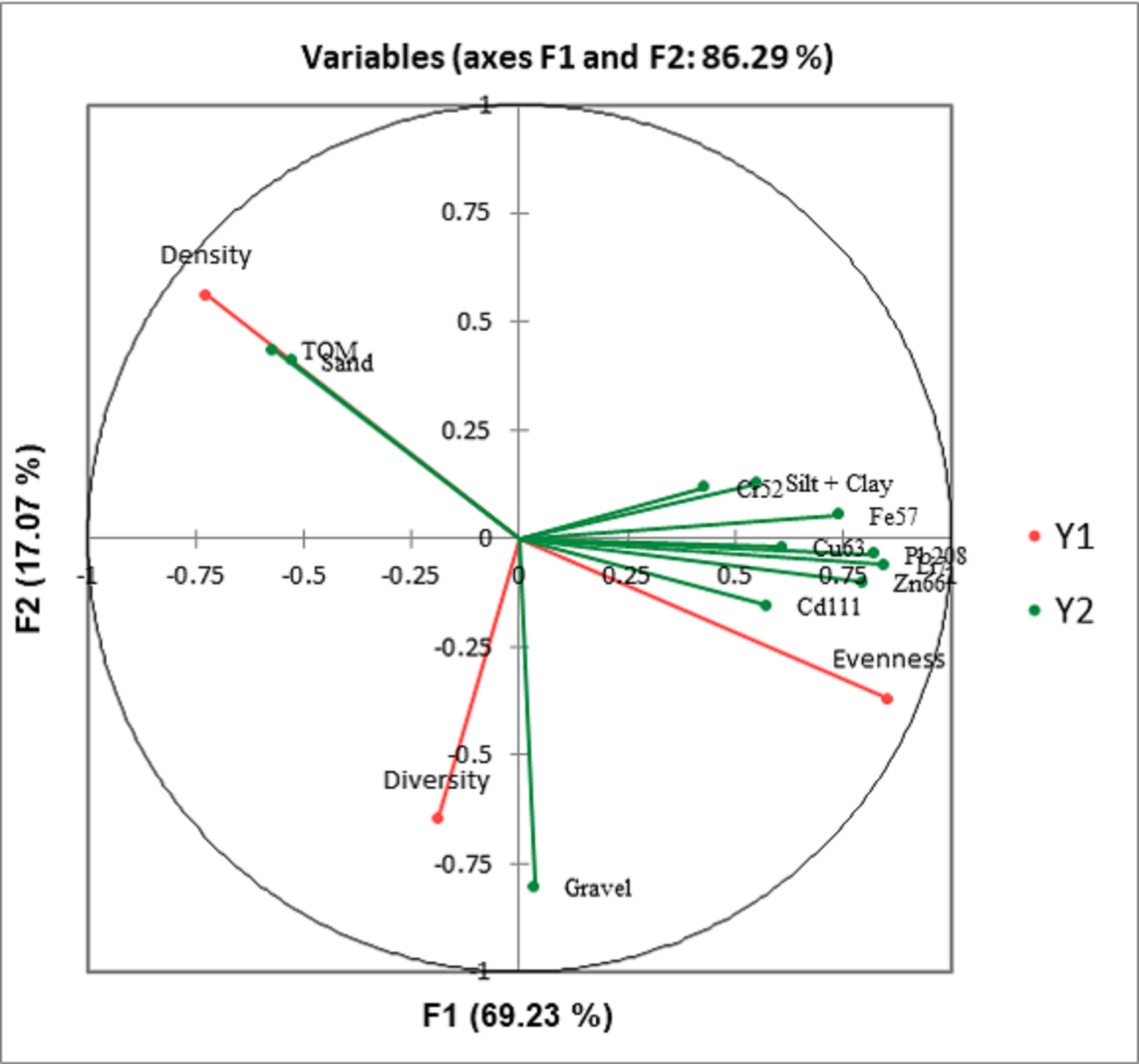
Canonical correlation analysis (CCA) plot showing the relationship between the benthic communities (density, diversity, and evenness) and the ecological parameters (grain size, total organic matter, and heavy metals).

Canonical correlation analysis (CCA) showed that macrobenthos density strongly correlates to TOM and sand. Still, it negatively correlated with gravel, heavy metals, and silt + clay (Fig. 8). The evenness is strongly correlated to heavy metals and gravel, but negatively correlated with TOM and sand. The macrobenthos diversity is closely related to gravel but negatively correlated with silt + clay (%) and TOM.

**Figure 9:**
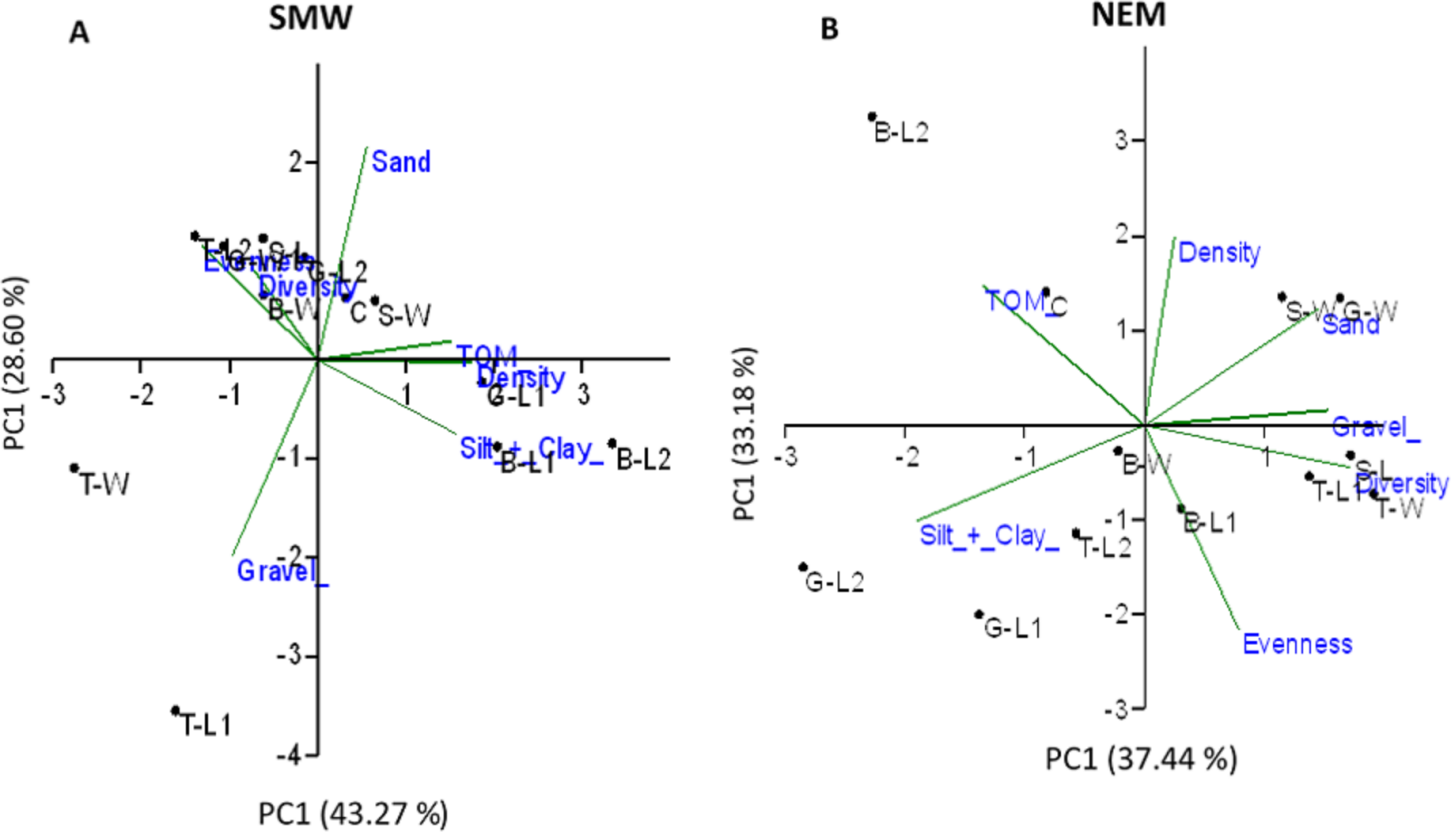
Principal component analysis (PCA) ordination showing sampled stations clustering based on a Euclidean distances matrix considering five environment parameters and three biotic assemblages in (A) SWM and (B) NEM.

**Figure 10:**
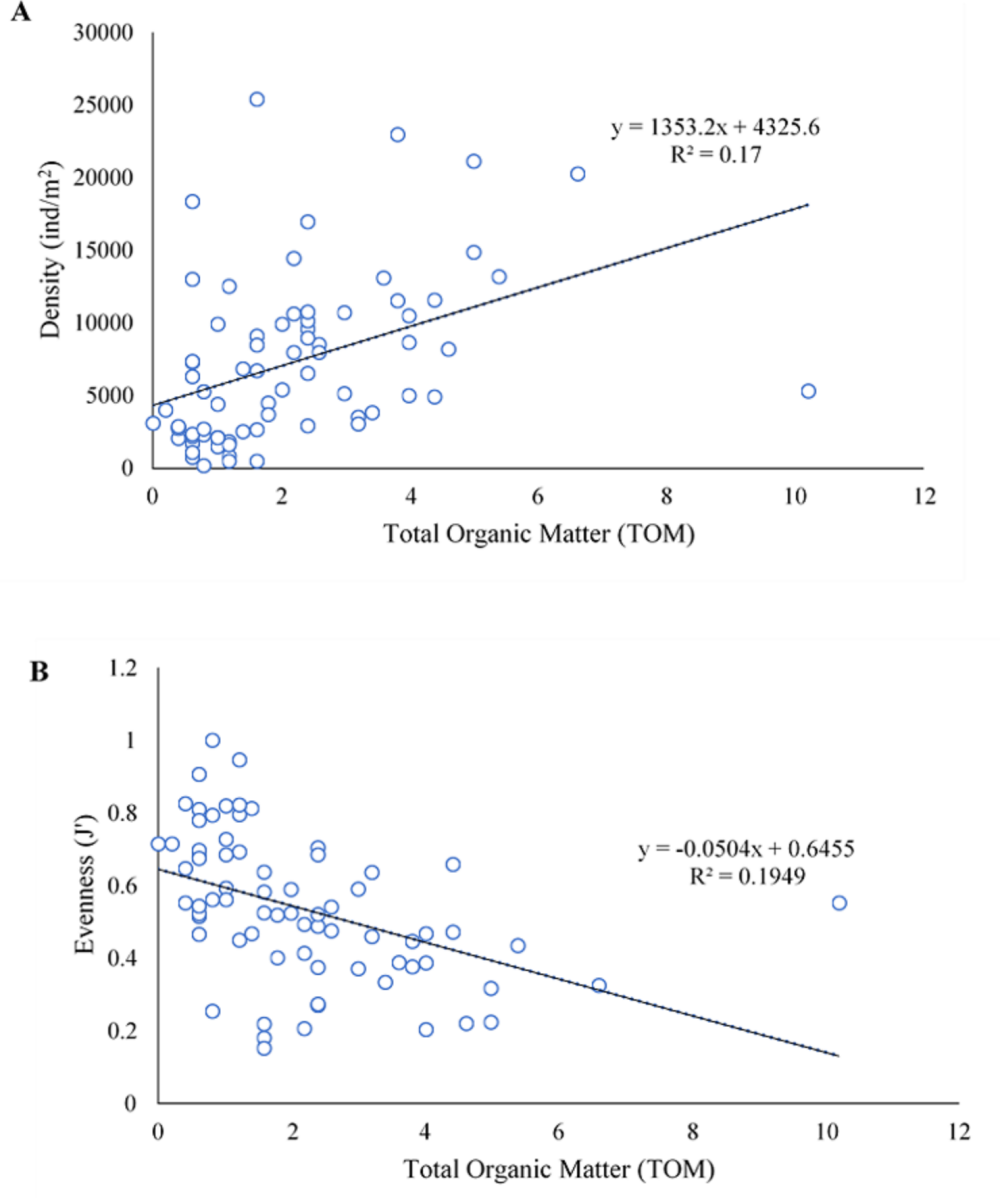
The linear negative relationship between density (ind./m^2^) (A) and evenness (J’) (B) of macrobenthos from Kuala Nerus coastal area, with total organic matter (TOM) (%).

In SWM, PC1 and PC2 accounted for 71.87% of the sample’s variation (Fig. 9A). PC1 showed that sand, TOM, and silt + clay were the most significant factors on axis 1 (total variance explained (TVE): 43.27%). The diversity and evenness of macrobenthos were negatively correlated with silt + clay, while the density of macrobenthos was strongly correlated with TOM. Gravel, the most significant factor on axis 2 (TVE: 28.60%), was negatively correlated to sand. In NEM, PC1 and PC2 accounted for 70.62% of the sample’s variation (Fig. 9B). PC1 showed that sand and gravel were the most significant factors on axis 1 (TVE: 37.44%), while TOM and silt + clay were the most significant factors on axis 2 (TVE: 33.18%). The density of macrobenthos was positively correlated with silt + clay, while the evenness of macrobenthos was positively correlated with TOM. The diversity of macrobenthos was strongly correlated to gravel. The output from MLR and model summary identified TOM (%) as the most significant predictor for macrobenthos’ density, evenness and composition (Fig. 10).

## Discussion

From one standpoint, the findings of this study suggest that the benthic communities associated with CDS will continue to change over time, regardless of the season. Burt, Bartholomew & Sale (2011) suggested that the benthic communities related to breakwaters continue to change over more than 31 years. As the breakwater age increases beyond 31 years, the community structure will converge toward the natural reef. However, the overall benthic community will remain distinct as the presence of the breakwater will surely prevent the migration of a few slow-moving species, thus affecting the distribution of those biotas. In addition, these changes will continue to occur until a point of stability is reached, which may take at least ten years for the artificial structures to develop into an area comparable to natural reefs, where the entire organisms can fully adapt.

From another standpoint, the results showed a significant temporal difference in the benthic assemblage (density). This is consistent with the findings by Munari et al. (2011), who found that the benthic assemblage composition at the low-crested breakwater differs significantly depending on the season (October *vs.* June). The author observed a significant difference at the seaward sites but none at the landward side of the structure between both seasons. The finding contrasts with this study, which revealed significant differences in benthic assemblages between seasons and seaward and windward sites. However, within the same season, such as in June of summer, the benthos community from the study by Munari et al. (2011) showed a significant difference between seaward and landward sites.

According to Parisi et al. (1985), the annual cycle of the cold season in the northern Adriatic Sea resulted in massive mortality of the benthic community, with population density then spiking drastically in summer for new recruitment. This scenario is similar to this study, which revealed the highest population of macrobenthos during SWM and a lower population during NEM. The structure of infaunal assemblages in summer, as in SWM, is much more similar to a biologically controlled one, owing to the stable hydrodynamic regime in the area. The reduction in hydrodynamic stress has essentially increased the organic matter content (food sources) and finer sediments in the area, causing a seasonal increase in species richness. The hydrodynamic regime of the area, which acts as a key feature that influences the structure and composition of benthic assemblages, also influences the intense mortality of macrobenthos throughout the seasonal change. Various CDS types have added high complexity and fluctuation to the pattern of macrobenthos assemblages, contributing to more significant findings of the organisms.

In addition, Liu, Li & Zhang (2017) highlighted that the differences in the macrobenthos community in both seasons are potentially shaped by several environmental parameters such as grain size distribution, total organic matter (TOM) content, and the current pattern at the study sites. The installation of CDS has fairly compromised water current patterns, particularly at the bottom, which could have led to nearby alterations in the marine environment, including grain size, and indirectly organic matter content. These changes could have affected the distribution of several species, altering the macrofaunal assemblages in sandy bottoms near CDSs. In this study, the windward substations that were directly facing the sea, such as substations G-W, B-W, and T-W, exhibited a coarser grain size (ranging from medium to very coarse sand) with a lower density of benthic organisms (4,000 to 9,000 ind./m^2^). These results are highly supported by Wambrauw, Krey & Ratnawati (2019), who stated that sediment dominated by coarser sand possessed lower primary productivity, negatively affecting organisms in that particular habitat. Carugati et al. (2018) also reported similar findings in which the grain size distribution analysis showed that sand was the dominant fraction in the wave-exposed area of the seaward side (approximately 90% of sand and 10% of pelite, on average).

According to different CDS systems represented by stations, the large groyne structure of station 1 harbors a significantly higher density of macrobenthos. This finding agrees with a study by Walker, Schlacher & Thompson (2008), which highlighted that the depositional side of groyne in Southern Queensland (Australia) exhibited a higher abundance and biodiversity of macrofaunal species. The lateral groyne has successfully prevented water circulation to a larger extent, creating a muddy pool that has supported a vast array of macrobenthos in the area (Becchi et al., 2014).

Moreover, the landward substations of the left and right sides of the CDS, such as substations G-L1, G-L2, B-L1, B-L2, T-L1, and T-L2, are mostly dominated by very fine sand regardless of the seasons, resulting in higher macrobenthos ranging from 3,900 to 16,000 ind./m^2^. This result agreed with a study by Masucci, Acierno & Reimer (2020), which identified the presence of finer sediment on the landward side of the wall (Breakwater-Back –fine sand, moderately sorted, very coarse skewed) possibly because of lower water energy levels. Theoretically, the fine-sand communities received sufficient and significant concentrations of nutrients and benefits from the primary production of the microphytobenthos (Hope et al., 2020).

This study demonstrated that the substratum of those stations was dense and hard, with an average grain size of approximately 150 µm and more frictional surfaces per unit volume than in the coarse sand. Conceptually, fauna must use enormous energy to penetrate or move in the substratum, causing them to simply stay just below the interface (Wiesebron et al., 2021). This concept could allow more macrobenthos from those stations to be documented in this study. This finding is also consistent with the study by Bertasi et al. (2007) and Munari et al. (2011), which stated that the number of taxa found on landward sheltered sites was significantly higher than the exposed sites, with a dominance of bivalves (50.8%) and polychaetes (43.3%). The breakwater barriers successfully created pools where the environmental conditions are more favorable for species to thrive, rather than the harsher environment on the outer side.

Another factor that highly influences macrobenthos distribution is the speed and pattern of water currents, causing waves to lap over the sediment. Concisely, the Terengganu current movement is governed by a monsoonal wind system in which the wind at the east coast of Peninsular Malaysia prevails from northeasterly during the NEM (speed of 10–20 knots, reaching up to 30 knots in some areas) and southwesterly during the SWM (speed of average 15 knots) (Kok et al., 2017). Meanwhile, the current moves northward during the NEM and southward during the SWM, depending on the intensity of the prevailing wind (Daud, Akhir & Husain, 2016). According to Ling et al. (2019), the current velocities toward the north of the Sultan Mahmud airport runway are higher during the pre-NEM season (average speed of 0.3 m/s). This speed is sufficient to move the bottom sand, consequently causing disturbance to the macrobenthos distribution during the NEM.

Theoretically, as the wave is always present on the seabed and fairly controlled by the CDS, there will be constant water movement through the sediment and interstitial space. Therefore, the heaviest particles will settle first along the coast, and the finest ones are carried the furthest, adding to the muddy bottoms (López, 2017). This granulometry concept is strongly associated with currents, in which the strongest current usually occurs in shallow waters with coarse sand, moderate currents over fine sand, and much slower currents over deep mud. This study demonstrated that the front side of the CDS has the strongest wave energy hit. The hard structure successfully broke the unstable area dominated by coarse sand and washed away the biota living in the vicinity. As a result, the macrobenthic community in these areas is lower than the stable areas of the backside of the CDS.

In addition to the immediate removal of biotas in an unstable environment, these scenario patterns will directly influence the deposit of organic matter in those areas, which is inversely proportional to the strength of the currents (Reyes & Crisosto, 2016). The percentage of organic matter is lower in the coarse sand of shallow areas and strong currents, and higher in the muddy area of greater depths and slower currents. These are the baseline conditions that define the distribution of fauna on the sea floor. This concept is consistent with the finding from this study, which demonstrated that the percentage of organic matter is lower (up to 3.1%) during the NEM season of stronger water currents than during the SWM season (6.33%) of weaker water currents. Regardless of the seasons, the percentage of organic matter was higher at landward stations (*e.g.*, G-L1, S-L, B-L2, T-L1, T-L2, and C), which constituted mostly very fine sand. On the other hand, the windward stations exhibited a lower percentage of organic matter. This result is supported by Carugati et al. (2018), which stated that the highest value of TOM at the sheltered sites could be related to the different breakwater designs that reduce the hydrodynamic export to the open sea, thus increasing the accumulation of organic matter in those areas.

In this study, the lowest average number of individuals and densities at station 3 during both seasons is attributed to the strong water current movement in that area. According to Zulfakar et al. (2020), some areas still receive high current speeds (*e.g.*, station 3) even though the presence of coastal structures successfully reduced the intensity of incoming currents in most areas along the Kuala Nerus coast. Although no recirculation of water current was observed, the current passing through the semi-enclosed jetty-type breakwater (station 2) directly hit the nearshore area, causing the disappearance of tombolo features behind the southernmost parallel breakwater (station 3). This theory could be the main factor for the least amount of macrobenthos found in those areas (B-L1, B-W, and B-L2) during both seasons. In addition, the coarser sand of lower productivity, which dominated the substations, contributed significantly to the lowest density of organisms in those areas.

Regarding the lower seasonal concentrations of heavy metal that bind to the surface sediment of the CDS area of Kuala Nerus, the allowable concentration alone does not influence the organisms’ diversity, composition, and assemblage pattern. Heavy metals introduced from various possible sources such as sewage and industrial effluents, brine discharge, agriculture activities, and coastal development could be transported by prevailing currents along the coastal area of Kuala Nerus. In addition, as the CDS area is dominated by sandy type substrate (very fine, medium, and coarse sands), the heavy metals did not absorb well onto the sand fraction of the smaller surface area. Clay, fine, and very fine silt particles with a larger surface area to volume ratio provide broader and more active sites for the absorption of heavy metals. These minute particles are better able to form large aggregates of metals, bound together by electrochemical force and organic matter than the sandy particles (Yunus, Zuraidah & John, 2020). This supports the lower seasonal concentrations of heavy metals in CDS areas of Kuala Nerus, which are dominated by sand.

The highest species composition of Mollusca recorded in this study was expected, given that Mollusca, particularly the Bivalvia, are common members of the early benthic community and generally of the highest dominance over the first several years of artificial structures (Wanninger & Wollesen, 2019). Notably, the Mollusca cover often peaks between 3.5 and 5.5 years and declines on older structures, likely due to growing coral cover in those areas. This phylum proved that the organisms demonstrate strong adaptation and survival skills, leading to a higher tolerance to the biological and chemical changes produced by the SWM. As the coastal structures in the study are also relatively wave-exposed, it suggests that the phylum is highly resilient to physical stressors (Fortunato, 2015). The intrinsic (*e.g.*, reproduction and stress resistance) and extrinsic (*e.g.*, habitat availability and species interactions) characteristics of the Mollusca also contributed to the most dominant phylum found in the stations (Airoldi, 2000).

## Conclusions

This study demonstrated that CDS plays a crucial role in controlling the distribution and composition of macrofaunal in the coastal areas of Kuala Nerus. However, macrobenthos composition fluctuates as the areas are not yet in an equilibrium state. The assemblages fluctuate more under the monsoonal system, with a higher macrobenthos composition in the SWM than that in the NEM. In addition, the CDS system associated with the monsoonal season has governed the hydrodynamics and nearshore sedimentary processes in the Kuala Nerus coastal areas.

In brief, as the benthic macrofauna reaches the nearshore area with the existence of coastal structures, the longshore currents of different seasons have laterally redistributed them, leading to significant differences between both seasons. The findings of this study contributed to the existing literature, particularly for Malaysian records, highlighting that coastal developments such as artificial structures can have important ecological benefits and positive impacts on the local marine ecosystem.

Notably, this study was conducted at a limited spatial scale of Kuala Nerus coasts and thus cannot be extrapolated to represent all CDS in Malaysia. The results may vary depending on CDS’s locations, designs, materials, and years of installation. Therefore, continuous studies are necessary due to the lack of data and information on CDS’s long-term effects and “footprint” on macrobenthos composition, particularly in Malaysia. The current results from this short-term experiment may not accurately reflect the outcomes observed in the longer term.

##  Supplemental Information

10.7717/peerj.16203/supp-1 Supplemental Information 1 Raw data on the total number of macrobenthos in Kuala Nerus, Terengganu

## Additional Information and Declarations

### Data Availability

The following information was supplied regarding data availability: The raw data is available in the Supplemental Files.
